# Evaluation of five treatments to control intestinal parasites in sheep in Ayapango, state of Mexico

**DOI:** 10.14202/vetworld.2016.1233-1237

**Published:** 2016-11-12

**Authors:** Rafael Heredia, Emma Aguilar, Camilo Romero, Linda Bautista, Germán Mendoza

**Affiliations:** 1 Department of Agricultural Sciences and Natural Resources, University Center UAEM Amecameca, Autonomous University of Mexico State, Mexico; 2 Department of Veterinary Medicine, University Center UAEM Amecameca, Autonomous University of Mexico State, Mexico; 3 Department of Veterinary Medicine, Research Academician of Animal Health, University Center UAEM Amecameca, Autonomous University of Mexico State, Mexico; 4 Department of Agricultural and Animal Production, Autonomous Metropolitan University Xochimilco, Mexico City, Mexico

**Keywords:** anthelmintics, control, intestinal parasites, sheep

## Abstract

**Aim:**

Intestinal parasites are one of the most common problems in sheep production systems. However, the strategies used to eliminate these parasites have not yielded satisfactory results. Therefore, the aim of this study was to determine the effect of five anthelmintics (with different active ingredients) on the parasite load in sheep.

**Materials and Methods:**

In this study, 107 Rambouillet breed sheep were randomly assigned to five groups. Next, fecal samples were taken directly from the rectum and sent to the laboratory for analysis. We then dewormed each group of sheep using different anthelmintic products: Ivermectin 1%/clorsulon 10%, levamisole 12%, closantel sodium 5%, ivermectin 10%, and closantel 5%/albendazole 3.75% with a dosage corresponding to each sheep. At 15 days post-treatment, we took fecal samples and performed a coproparasitoscopic study, using the Faust flotation technique to assess the presence or absence of parasite eggs and the McMaster technique to quantify eggs.

**Results:**

Ivermectin/clorsulon was more effective in eliminating parasites than other anthelmintics used, especially in *Haemonchus* spp.

**Conclusion:**

The results of this study indicate that using ivermectin/clorsulon decreases the number of eggs in feces and is one alternative in controlling parasites in sheep, leading to a reduction in the incidence of health problems, and consequently, improved productivity.

## Introduction

Intestinal parasites such as *Haemonchus* spp., *Moniezia* spp., *Ostertagia* spp., and *Chabertia* spp. are one of the most common health problems affecting sheep (especially young animals), and which seriously affect their health, causing poor weight gain, thus making them susceptible to secondary diseases that may eventually cause death [1]. Such problems incur significant economic losses for producers. Grazing management is a factor that predisposes sheep to acquire parasites [2]. The use of anthelmintics in cattle has reduced the impacts of gastrointestinal nematodes [3]. Control strategies involve actions directed to the host, to eliminate parasites and thereby reduce the contamination of pastures [4].

Frequent use of broad spectrum anthelmintics has greatly increased the prevalence of anthelmintic resistance [5], which is why we need studies that provide insight into the types of parasites present in sheep to determine the antiparasitic strategy that should be employed.

In Mexico, products derived from benzimidazole and macrocyclic lactones are the most frequently used in cases of intestinal parasites, given their broad spectrum of action. High parasitic resistance to benzimidazole has been observed, but this is not the case with macrocyclic lactones [6]. Ivermectins are semisynthetic macrocyclic lactones derived from avermectin. Using ivermectin in cattle in Mexico depends on the farmer’s and/or veterinarian’s perception of the likelihood of parasite infestation of the cattle, which tends to be seasonal [7]. The State of Veracruz, as one of the main cattle producers in the country, consumes large amounts of ivermectin, which could also be the case in other states of México [8]. The constant use of levamisole has led to reduced efficiency (30%) in the control of gastrointestinal nematodes (*Haemonchus contortus* and *Cooperia curticei*) in hair sheep. Moreover, the combination of levamisole with ivermectin is associated with anthelmintic resistance, and closantel/albendazole also leads to resistance, tested using the larval motility test *in vitro* in a study conducted in the state of Chiapas in Mexico [9]. Albendazole, a benzimidazole derivative, is an anthelmintic drug that has been widely used around the world for decades in cattle and small ruminants [3].

These treatments have been selected for use widely by Mexican farmers, sometimes in excessive or inadequate doses. The objective of this study was to evaluate these five treatments to control intestinal parasites in sheep in Ayapango, State of Mexico.

## Materials and Methods

### Ethical approval

The experiment was approved by Institutional Animal Ethics Committee.

### Selection of animals

For this study, 107 Rambouillet breed sheep from the Ayapango, State of Mexico, were randomly selected in the autumn-winter. Specifically, this included 105 females and two males, with an age mean of 21.7 months. The flock was sent out daily for grazing, not separated by fences. Sheep were identified by ear tags, and all sheep had gone more than 8 months without deworming.

### Treatments

Sheep were organized into five groups.

#### Group 1

Consisting of 18 female sheep dewormed with ivermectin 1%/clorsulon 10% at a dosage of 2.0 mg/kg body weight, administered subcutaneously.

#### Group 2

Consisting of 21 female and one male sheep dewormed with 12% levamisole at a dosage of 6.0 mg/kg, administered via deep intramuscular injection.

#### Group 3

Consisting of 26 females and one male sheep dewormed with 5% closantel sodium at a dosage of 15 mg/kg, administered orally.

#### Group 4

Consisting of 18 female sheep dewormed with ivermectin 1% at a dosage of 4.0 mg/kg, administered subcutaneously.

#### Group 5

Consisting of 22 female sheep dewormed with closantel 5%/albendazole 3.75% at a dosage of 10 mg/kg, administered orally.

The use of a control group was not considered because the five treatments were evaluated and compared according to the difference in the number of eggs pre- and post-treatment.

After application of the treatments, sheep were placed in the same corral, and always had the same diet by grazing together in the same place.

### Analysis of fecal samples

From these individuals, we took fecal samples directly from the rectum, which were identified and stored in plastic bags for later analysis. Sample processing was performed in the laboratory of parasitology and microbiology of the Amecameca University Center of the Autonomous University of the State of Mexico, 15 days after the drugs were administered, fecal samples were taken and analyzed by a coproparasitoscopically using the Faust flotation technique with 2.04 g/mol zinc sulfate. McMaster chambers were used for subsequent egg counting and the determination of parasite load [10-12].

### Statistical analysis

The results were analyzed using a Kruskal–Wallis non-parametric test because the groups did not have the same number of animals, and the data did not present a normal distribution.

## Results and Discussion

Table-1 shows that there was a statistically significant difference (p<0.0001) between number of eggs of *Toxocara* spp. before and after administration of 2.0 mg/kg ivermectin/clorsulon. In the case of *Haemonchus* spp., there was also a significant difference (p=0.0279) between the number of eggs before and after the same treatment. The same was observed for *Ostertagia* spp. (p<0.0001) and *Chabertia* spp. (p=0.0036). In contrast, there was no difference in the number of eggs of *Moniezia* spp. (p=0.1271) before and after treatment.

**Table 1:** Mean number of eggs pre- and post-treatment with ivermectin/clorsulon in Group 1.

Parasite (eggs)	n	Number of eggs* pre-treatment	Number of eggs* 15 days post-treatment	χ^2^	p**
*Toxocara* spp.	18	25.277	11.722	18.043	<0.0001
*Haemonchus* spp.	18	21.166	15.833	4.832	0.0279
*Moniezia* spp.	18	20.111	16.888	2.327	0.1271
*Ostertagia* spp.	18	25.305	11.694	17.676	<0.0001
*Chabertia* spp.	18	22.777	14.222	8.450	0.0036

* Mean number of eggs,

** p≤0.05

In the comparison shown in Table-2, the number of *Ostertagia* spp. eggs before and after treatment showed a statistically significant difference (p<0.0001). There was a significant difference in the number of eggs of *Chabertia* spp. (p<0.0001) after the administration of 6.0mg/kg of levamisole, while for *Toxocara* spp. there was no significant difference in the number of eggs pre and post-treatment (p=0.087). This was also true for *Haemonchus* spp. (p=0.141) and *Moniezia* spp. (p=0.084).

**Table 2:** Comparison of the number of eggs pre- and post-treatment with levamisole in Group 2.

Parasite (eggs)	n	Number of eggs* pre-treatment	Number of eggs* 15 days post-treatment	χ^2^	p**
*Toxocara* spp.	22	24.727	20.272	2.928	0.087
*Haemonchus* spp.	22	24.068	20.931	2.161	0.141
*Moniezia* spp.	22	24.954	20.045	2.983	0.084
*Ostertagia* spp.	22	31.250	13.750	21.856	<0.0001
*Chabertia* spp.	22	31.772	13.227	25.820	<0.0001

* Mean number eggs,

** p≤0.05

Table-3 shows that treatment with 15 mg/kg closantel sodium resulted in a statistically significant difference in the number of eggs of *Toxocara* spp. (p<0.0001), *Ostertagia* spp. (p<0.0001), and *Chabertia* spp. (p<0.0001), while no statistically significant difference was observed for *Haemonchus* spp. (p=0.060) or *Moniezia* spp. (p=0.1663).

**Table 3:** Comparison of the number of eggs pre- and post-treatment with closantel sodium in Group 3.

Parasite (eggs)	n	Number of eggs* pre-treatment	Number of eggs* 15 days post-treatment	χ^2^	p**
*Toxocara* spp.	27	35.555	20.444	16.022	<0.0001
*Haemonchus* spp.	27	30.518	24.481	3.537	0.060
*Moniezia* spp.	27	29.722	25.277	1.916	0.1663
*Ostertagia* spp.	27	40.314	14.685	37.314	<0.0001
*Chabertia* spp.	27	37.611	17.388	24.502	<0.0001

* Mean number of eggs,

** p≤0.0

Table-4 shows there was a statistically significant difference in the number of *Toxocara* spp. (p=0.0039), *Haemonchus* spp. (p=0.0019), and *Ostertagia* spp. (p=0.004) eggs after the administration of 4.0 mg/kg ivermectin, but for *Moniezia* spp. (p=0.0927) and *Chabertia* spp. (p=0.491), there was no significant difference in egg number.

**Table 4:** Comparison of the number of eggs pre- and post-treatment with ivermectin in Group 4.

Parasite (eggs)	n	Number of eggs* pre-treatment	Number of eggs* 15 days post-treatment	χ^2^	p**
*Toxocara* spp.	18	22.0	15.0	8.352	0.0039
*Haemonchus* spp.	18	23.527	13.472	9.628	0.0019
*Moniezia* spp.	18	20.416	16.583	2.827	0.0927
*Ostertagia* spp.	18	23.222	13.777	8.127	0.004
*Chabertia* spp.	18	19.333	17.666	0.472	0.491

* Mean number of eggs,

** p≤0.05

In the group treated with 10 mg/kg closantel/albendazole (Table-5), there was a statistically significant difference in the *Toxocara* spp. egg number (p=0.0007), in contrast to *Cooperia* spp. (p=0.129), *Trichostrongylus* spp. (p=0.177), *Oesophagostomum* spp. (p=0.664), *Haemonchus* spp. (p=0.263), *Trichuris* spp. (p=0.408), *Moniezia* spp. (p=0.767), and *Strongyloides* spp. (p=0.416), which showed no differences in egg numbers after treatment.

**Table 5:** Comparison of the number of eggs pre- and post-treatment with closantel/albendazole in Group 5.

Parasite (eggs)	n	Number of eggs* pre-treatment	Number of eggs* 15 days post-treatment	χ^2^	p**
*Toxocara* spp.	22	26.714	17.500	11.441	0.0007
*Cooperia* spp.	22	24.857	19.272	2.295	0.129
*Trichostrongylus* spp.	22	24.523	19.590	1.817	0.177
*Oesophagostomum* spp.	22	22.761	21.272	0.188	0.664
*Haemonchus* spp.	22	24.095	20.000	1.251	0.263
*Trichuris* spp.	22	23.523	20.545	0.684	0.408
*Moniezia* spp.	22	22.428	21.590	0.087	0.767
*Strongyloides* spp.	22	23.404	20.659	0.659	0.416

* Mean number of eggs,

** p≤0.05

The closurlon is use for treatment of flukes without effect in nematodes, although this drug combination is used for the treatment of flukes and nematodes, the coproparasitoscopic tests on samples from sheep treated with ivermectin/clorsulon demonstrated efficiency in removing most parasites, especially *Haemonchus* spp., one of the most commonly implicated parasites in weight loss in sheep [13]. The administration of these two drugs had a positive effect on sheep by reducing the number of various intestinal parasites [14]. In a study in rats infected with *Fasciola*, by Sibille *et al.*, they found that the combination of these anthelmintics in male mice had little efficacy unlike females where efficacy was higher, but in general, if there were flukes reduction post-treatment [15].

Sakhawat *et al.*, in 1997, they reported that levamisole had 100% efficiency that no nematode eggs were recovered from animals treated [16]. In more recent studies have shown that levamisole administration in sheep does not reduce the majority of intestinal parasites [17]. *Haemonchus* spp. has developed resistance due to the regular use of this anthelmintic in sheep [18], this is consistent with our results because the number of eggs of *Toxocara* spp., *Haemonchus* spp., and *Moniezia* spp. not decreased, with this treatment, although the number of eggs to *Ostertagia* spp. and *Chabertia* spp. decreased significantly, which means that, the parasites aforementioned (*Toxocara* spp., *Haemonchus* spp. and *Moniezia* spp.) they were resistant to this drug.

Closantel sodium has efficacy against strongyle populations which are resistant to ivermectin [19], Similarly, the results to this study, as can be seen in Table-3 where we see that; *Toxocara* spp., *Ostertagia* spp., *Chabertia* spp., if they decrease significantly, but it is not very useful against *Haemonchus* spp. as describe Jabbar *et al.*, in 2013 [20].

In this research ivermectin shows excellent nematicide efficacy, which is consistent with that reported con Muñoz *et al.*, but also it mentions that, this has diminished over time, especially in the genera *Trichostrongylus* and *Haemonchu*s spp., which have developed resistance [21]. This can happen for various reasons, including the application of inadequate doses of ivermectin, the absence of a timetable for deworming according to the cycles of the parasites and their seasonality, as well as the introduction of animals from areas where resistance has occurred [22].

In our results find that a combination of closantel/albendazole, it was only effective against *Toxocara* spp. and it had no effect on *Cooperia* spp., *Trichostrongylus* spp., *Oesophagostomum* spp., *Haemonchus* spp., *Trichuris* spp., *Moniezia* spp. and *Strongyloides* spp. in our study, which coincides with that reported by other authors who mention that; closantel has demonstrated ineffectiveness in deworming programs, which is why it is suggested to alternate it with other anthelmintics to improve its effect [23]. Meanwhile, albendazole has a prolonged effect and it is most effective when treatment is administered for an extended period [24]. Thus, even in combination, these two anthelmintics are not a good alternative for deworming.

## Conclusion

Misuse and uncontrolled anthelmintic administration in sheep production systems have led to the development of resistance in some parasites. Therefore, it is important to monitor this problem and parasite infestation triggers nutritional, health, and economic problems for producers. The results of this study show that effective control of intestinal parasites in sheep can be achieved using a combination ivermectin and clorsulon, which reduces the incidence of intestinal parasitosis.

## Authors’ Contributions

CR and EA planned and designed the study. EA collected samples. EA analyzed samples. LG, RH, and GM analyzed the data and provided technical support. EA and RH prepared the manuscript with guidance from the other authors. All authors read and approved the final manuscript.
